# Implementation of maternity protection legislation: Gynecologists’ perceptions and practices in French-speaking Switzerland

**DOI:** 10.1371/journal.pone.0231858

**Published:** 2020-04-30

**Authors:** Alessia Abderhalden-Zellweger, Isabelle Probst, Maria-Pia Politis Mercier, Brigitta Danuser, Pascal Wild, Peggy Krief

**Affiliations:** 1 HESAV School of Health Sciences, HES-SO University of Applied Sciences and Arts Western Switzerland, Lausanne, Switzerland; 2 Occupational Health and Environment Department (OHED), Center for Primary Care and Public Health (Unisanté), Epalinges, Switzerland; 3 INRS Research and Studies Management, Vandoeuvre les Nancy, France; Erasmus Medical Center, NETHERLANDS

## Abstract

**Background:**

In several countries, maternity protection legislations (MPL) confer an essential role to gynecologist-obstetricians (OBGYNs) for the protection of pregnant workers and their future children from occupational exposures. This study explores OBGYNs’ practices and difficulties in implementing MPL in the French-speaking part of Switzerland.

**Methods:**

An online survey was sent to 333 OBGYNs. Data analysis included: 1) descriptive and correlational statistics and 2) hierarchical cluster analysis to identify patterns of practices.

**Results:**

OBGYNs evoked several problems in MPL implementation: absence of risk analysis in the companies, difficult collaboration with employers, lack of competencies in the field of occupational health. Preventive leave was underused, with sick leave being prescribed instead. Training had a positive effect on OBGYNs’ knowledge and implementation of MPL. Hierarchical cluster analysis highlighted three main types of practices: 1) practice in line with legislation; 2) practice on a case-by-case basis; 3) limited practice. OBGYNs with good knowledge of MPL more consistently applied its provisions.

**Conclusion:**

The implementation of MPL appears challenging for OBGYNs. Collaboration with occupational physicians and training might help OBGYNs to better take on their role in maternity protection. MPL in itself could be improved.

## Introduction

From the public health and health promotion perspectives, pregnancy-related care is essential given that the health of future generations is already determined by babies’ lives within the womb [1]. Although the international medical literature agrees that work in and of itself poses no risk to pregnancy [2, 3], several publications [2–12] show that occupational exposure (biological, chemical) or arduous activity (physical, psychological) can affect pregnancy outcomes (miscarriage, preterm birth, small for gestational age) and child development (malformation, cognitive faculties).

### The Swiss labor market

Switzerland has a liberal labor legislation, with little state intervention [13]. It confers companies a lot of decisional power and a relatively small amount of control and sanctions [13].

The Swiss labor market is characterized by a high rate (76%) of companies in the tertiary sector (mostly service activities, with a predominance of companies involved in the healthcare sector) and of small companies: 90% have less than 10 employees and 8% between 10 and 49 employees [14]. The study has been conducted in French speaking cantons of Switzerland (Vaud, Valais, Genève, Fribourg, Jura and Neuchâtel). This part of Switzerland comprises about ¼ (2 211 571 inhabitants) of the total Swiss population. Approximately, 534 953 women of reproductive age (between 15 and 50 years) live in this region of Switzerland [15]. In 2017, when the study has been conducted, 26 526 births were registered in the six French speaking cantons [16]. In this area, the three most dominant economic sectors for women are: the healthcare, the retail sector and the education [17].

**Table 1** gives relevant characteristics of the Swiss labor market [18, 19].

**Table 1:** Relevant characteristics of the Swiss labor market and their comparison with the European average.

	Switzerland	European average
Women engaged in a professional activity in the 25 to 54 age group [18]	82.2%	63,4%
Working hours per week [19]	42 hours	39 hours
Companies that regularly evaluate the occupational risks in the workplaces [19]	45,2%	74%
Companies that have formal representations of employees (i.e. trade union) [19]	36.8%	51%
Companies that refer to an occupational physician [19]	12.1%	61.9%

### The Swiss legal framework concerning the protection of pregnant workers

Switzerland has introduced the Ordinance on Maternity Protection at Work [20, 21] (*OProMa*) in order to protect the health of pregnant women from occupational exposures, while allowing them to continue to work in suitable conditions. The purposes of this Ordinance are in conformity with International Labour Organization Recommendation 191 on maternity protection [22]and with the Council Directive 92/85/EEC of 19 October 1992 [23]. The literature review conducted by Probst, Zellweger [24]provides a comparison of features of maternity protection legislation in different countries.

The OProMa stems from the Swiss Labor Law (LTr, section 35), which defines a general framework for the protection of the health of pregnant workers [20, 25]. This Ordinance sets out which types of jobs are considered dangerous or arduous (see **Table 2**), the processes to be put in place to counter these risks, and the responsibilities of all the actors involved [26].

**Table 2:** Risk's activities within the meaning of the OProMa [27].

Article of the legislation	Types of working conditions and activities that are considered to be dangerous or arduous for pregnant employees under the OProMa	Detailed description if applicable
OProMa art. 7	Shifting heavy loads	Not more than 5 kg after 6th month of pregnancy
OProMa art. 8	Exposure to heat, cold, and humidity	Between -5°C and 28°C
OProMa art. 9	Movements and postures generating an early fatigue or other tough conditions such as vibrations, shocks and bumps	-
OProMa art. 10	Exposure to micro-organisms	-
OProMa art. 11	Noise exposures	Not-admitted ≥ 85dB(A)
OProMa art. 12	Ionizing and non-ionizing radiation	Limit values are described in the Radiological Protection Ordinance (RPO).
OProMa art. 13	Exposure to dangerous chemicals	-
OProMa art. 14	Constraining working-time organization	Not more than 3 night shifts. Night work is prohibited for dangerous activities within the meaning of Articles 7 to 13.
OProMa art. 15	Piecework and/or activities at predetermined work-rate without the possibility of flexibility from the pregnant employee.	-
OProMa art. 16	Work in overpressure or in workplaces with oxygen-reduced atmosphere	-

The OProMa confers an essential role on gynecologist-obstetricians (OBGYNs); midwives can and do monitor pregnancies in Switzerland, nevertheless, they don’t have a legally defined role in the OProMa.

During pregnancy consultations, OBGYNs must verify whether their patients are exposed to any occupational activities banned under the OProMa. If they are, OBGYNs must ask employers for a risk analysis (RA)–to be conducted by an appointed health and safety specialists, usually an occupational health physician–and decide on whether the expectant mothers can safely continue their jobs. In the absence of an RA, but in the presence of presumed hazards, OBGYNs should prescribe a certificate of preventive leave according to the precautionary principle. The employer must continue to pay the pregnant employee 80% of her salary. Preventive leave is different from sick leave, which implies that the patient presents with a pregnancy-related pathology. Sick leave is financed either directly by the employer or by the employer’s loss-of-income insurance whereas preventive leave is entirely financed by the employer.

Regarding other rights of pregnant workers, unlike most of the other industrialized countries, Swiss legislation does not provide any prenatal leave [28]. The Federal Act on compensation for loss of income by reason of service or maternity grants workers the right to a paid maternity leave of 14 weeks (or 98 days) to be taken all at once after childbirth [29]. Workers are protected against dismissal during the whole pregnancy and 16 weeks after childbirth, except for those on trial period [30].

### Disparities between OBGYNs’ practices and those provided for by legislation

Numerous studies have demonstrated shortcomings in the application of countries’ MPL, in different contexts and at different levels [24]. Furthermore, in contexts where OBGYNs play a role in the application of MPL, it has been shown that they do not necessarily feel competent to judge working conditions and pregnant women’s ability to work [31, 32]. A case study in Switzerland [33]and a large-scale study in Poland [34]showed that if there were suspicions of occupational risk, some OBGYNs wrote sick leave certificates (not provided for by the respective laws) instead of preventive leave certificates. Certain studies [32, 35–39] have suggested that granting sick leave could be one means adopted by OBGYNs, or requested by pregnant employees themselves, to react to potential workplace dangers.

Considering the difficulties facing its application, and with regard to the essential role given to OBGYNs in the OProMa, it seemed important to study their practices within the Swiss context.

The study aims to:

- Analyze the extent to which OBGYNs implemented MPL in French-speaking Switzerland.
- Highlight the barriers OBGYNs identified in their daily practice vis-à-vis MPL implementation.
- Explore the existence of OBGYNs’ different types of practices towards maternity protection.

## Materials and methods

### Ethics statement

The Human Research Ethics Committee of the Canton Vaud (CER-VD) has certified that the research study protocol associated with this study falls outside of the field of application of the Swiss Federal Act on Research Involving Humans.

The participation in the study was voluntary. All the participants included in the study were informed about the research objectives and the standards of confidentiality regarding the use of the data.

In the email sent to the OBGYNs, participants were informed about the objectives of this study and the confidentiality regarding the use of the gathered data. By accepting to fulfill the questionnaire on a voluntary base, the OBGYN agreed on the intended use of their data.

### Study population

The target population of OBGYNs working in public or private practices was identified from the cantonal registries of OBGYNs in the French-speaking part of Switzerland. All OBGYNs for whom valid email addresses could be obtained (n = 333) were contacted.

### Data collection

An electronic questionnaire addressing OBGYNs’ knowledge and perception about MPL was created and provided to the study population through email addresses. In the email sent to the OBGYNs, participants were informed about the objectives of this study and the confidentiality regarding the use of the gathered data. By accepting to fulfill the questionnaire on a voluntary base, the OBGYN agreed on the intended use of their data.

Questions were developed based on the scientific literature and authors’ clinical experience. Three external evaluators have tested the survey–notably in order to check the readability of the questionnaire. An OBGYN also provided an expert opinion on the questionnaire.

The electronic format of the survey was chosen to reach a larger number of participants [40]. This format also allowed OBGYNs to have an ample response time (almost three months). From April to June 2017, we send four reminders only to the participants who did not answer at our questionnaire or whose entry of data was in progress in order to encourage a higher response rate. Data collection was closed the 30^th^ June 2017.

The response rate was 32% (n = 105). The question “Are pregnancy consultations part of your professional activity?” was used to filter this population: 93 OBGYNs answered *yes*.

### Data analysis

Data were analyzed statistically in two stages using STATA 15 software:

1. Simple descriptive and correlational statistics using Fisher’s exact test;
2. Hierarchical cluster analysis to generate types (*clusters*) of responses from the variables describing OBGYNs’ practices. The objective was to identify types of practice by grouping subjects who gave similar responses.

Types of practices were identified by asking OBGYNs how frequently during their consultations they asked questions about occupational health, requested an RA, prescribed sick or preventive leave, or gave patients advices on MPL. Their decisions on whether to contact employers or whether to refer patients to an occupational physician were also recorded.

The variables for the generation of clusters were chosen by consensus within the research team. Questions with overly high rates of missing responses were not retained for analysis.

## Results

### Simple descriptive statistics

The OBGYNs estimated that 22%, on the average, of their patients was carrying out an occupational activity that posed a risk to their pregnancy. According to the OBGYNs, the five most frequent risk activities were: heavy loads (90.9%), standing for long periods (79.6%), detrimental psychological atmosphere (78.4%), strained postures or movements (64.8%) and stressful job (53.4%) (see **Table 3**).

**Table 3:** Descriptive statistics and characteristics of participants.

		OBGYNs (n = 93)
Age: mean (sd)		50.1 (9.9)
Years of experience as a OBGYN: mean (sd)		19.6 (9.5)
Estimated percentage of patients facing an occupational risk: mean (sd)		22.2 (15.4)
Estimated percentage of risk analyses received for patients facing an occupational risk: mean (sd)		5.4 (15.9)
		**% (n)**
Perceived knowledge about MPL	None at all	1 (1)
	Some	31 (29)
	Fairly good	57 (53)
	Very good	11 (10)
“Often” or “always” ask questions about:	Profession	99 (89)
	Occupational risks	86 (78)
	Workplace conditions	85 (76)
	Satisfaction at work	66 (61)
The five most commonly risk activities encountered by OBGYNs during pregnancy consultation	Heavy loads	90.9 (80)
	Standing for long periods	79.6 (70)
	Detrimental psychological atmosphere	78.4 (69)
	Strained postures or movements	64.8 (57)
	Stressful job	53.4 (47)
Frequency with which OBGYNs requested a risk analysis from the employer when receiving a patient whose job entailed a risk to her pregnancy	Never/rarely	35 (30)
	Sometimes	37 (32)
	Often	13 (11)
	Nearly always/always	15 (13)
Contact with the employer of a patient whose work poses a risk to pregnancy		58 (50)
Reasons explaining no contact with employers in cases involving suspected occupational risk and the absence of a risk analysis	Refusal by the patient	48 (40)
	Time constraints	29 (24)
	Perceived lack of experience or competencies	26 (22)
	It is the occupational health physician’s responsibility	18 (15)
	I have never thought about it	14 (12)
	I have to maintain medical secrecy	13 (11)
Difficulties in contacting the employer		70 (35)
Reason explaining the difficulties in contacting the employer	Times constraints	50 (25)
	Employer unavailable	40 (20)
	Medical secrecy	14 (7)
Difficulties implementing OProMa with the employer		70 (35)
Reason explaining the difficulties in implementing OProMa with the employer	The employer ask for sick leave to be granted	97 (34)
	Absence of any risk analysis	66 (23)
	Lack of knowledge about employers’ obligations	60 (21)
	An underestimation of the occupational risks	54 (19)
	A lack of collaboration	54 (19)
	The employer claimed economic difficulties	23 (8)
Frequency of prescription of preventive leave during normal pregnancies	Never/rarely	36 (31)
	Sometimes	33 (28)
	Often	20 (17)
	Nearly always/always	11 (10)
Frequency of prescription of sick leave during normal pregnancies	Never/rarely	15 (13)
	Sometimes	28 (24)
	Often	40 (34)
	Nearly always/always	17 (15)
When OBGYNs prescribe sick leave instead of preventive leave, it is “nearly always” or “always” because of:	A request by the patient	60 (51)
	A lack of competency in the domain of occupational heath	34 (29)
	Habit	34 (29)
	A request by the employer	26 (22)
	Time constraints	18 (16)
Frequency with which advices on MPL are given to patients	Never/rarely	12 (10)
	Sometimes	26 (22)
	Often	31 (26)
	Nearly always/always	31 (26)
Referred patients to occupational health physicians in cases involving suspected or proven occupational risks		62 (53)
Reasons explaining referral to an occupational health physician	To carry out a risk analysis	87 (45)
	To manage the situation because I do not have the time	75 (39)
	To manage the situation because I do not feel that I have the competencies	65 (34)
	To protect myself legally	25 (13)
Reasons explaining non-referral to an occupational health physician	I do not know any occupational health physicians	74 (5)
	I did not think about it	35 (11)
	I can manage the situation myself	16 (5)
	I could not find any occupational health physicians available	13 (4)
OBGYNs who attended a training program on pregnant employees and the OProMa		51 (43)
Perceived usefulness of the training		93 (40)
MPL is an important means of protecting pregnant employees		98 (83)
MPL is too burdensome on employers		58 (50)
MPL is insufficient because it does not cover all female employees		90 (76)
MPL is insufficient because it does not cover all occupational risks		79 (67)
Prescribing preventive leave should be the responsibility of an occupational health physician		81 (69)
Prescribing preventive leave may adversely affect the patient, particularly on her return to work after maternity leave		82 (70)

The majority of OBGYNs (68%) thought that they knew the MPL “quite well” or “very well”. During their consultations, OBGYNs stated that they “often/always” asked questions about patients’ professions (99%), the existence of any occupational risks (86%), working conditions (85%), and job satisfaction (66%).

On average, OBGYNs estimated that they received employers’ RAs in only about 5% of cases where their patients had a job involving a maternity protection risk. This was despite the fact that, according to the legislation, employers should have carried out an RA before even hiring a woman. Furthermore, 35% of OBGYNs declared that they “never/rarely” requested an RA when receiving a patient whose job entailed a risk to her pregnancy.

Among OBGYNs who did contact employers (58%), the majority (70%) attested to having encountered difficulties in making contact, most notably through their own time constraints (50%) or because of employers’ unavailability (40%). They also claimed to have encountered complications when attempting to implement the OProMa with employers (70%). The main reasons mentioned for these difficulties were the absence of any RA (66%) and, above all, that employers asked for their employees to be put on sick leave (97%).

In cases involving a non-pathologic pregnancy and a proven risk, only 32% of OBGYNs “often/always” prescribed preventive leave, whereas 57% declared that they “often/always” prescribed sick leave at the patient’s request (60%), out of habit (34%), because of a perceived lack of competency in the domain of occupational health (34%), at the employer’s request (26%), or because of time constraints (18%).

The majority of OBGYNs (62%) said that they “often/always” gave their patients advices vis-à-vis MPL. Indeed, 62% of OBGYNs also affirmed that they referred their patients to an occupational physician if they suspected or identified an occupational risk. The main reasons for this were: the possibility to have an RA carried out by an occupational physician (87%), the OBGYNs need for support due to time constraints (75%), and the OBGYNs lack of competencies in the domain of occupational health (65%). On the other hand, the main reasons explaining why OBGYNs did not refer patients to an occupational physician were that they did not know one (74%) and that they had never thought about it (35%).

Of the OBGYNs questioned, 51% had undergone specific training on pregnant workers and the OProMa. Among them, 93% considered that the training had helped them in their daily practice.

Nearly all OBGYNs (98%) believed that MPL are an important tool for protecting pregnant workers. However, the majority (58%) thought that the legislation is too burdensome on employers, yet insufficient because of its failure to cover all workers (90%) and all occupational risks (79%). Some 81% of OBGYNs believed that prescribing preventive leave should be the responsibility of an occupational physician, and 82% feared that prescribing preventive leave might put the pregnant worker’s career at risk, particularly on return from maternity leave.

### Associations between variables

**Table 4** displays the significant associations between the main characteristics of OBGYNs and important items. We only show associations with a significance level of *p* < 0.05. The model includes simultaneously OBGYNs’ sex, years of experience, and whether they had undergone training on pregnant workers and the OProMa, with adjustment for place of practice (hospital environment, private practice, or both). We found no significant association between the canton of practice and the obtained outcomes.

**Table 4:** Significant associations between the main characteristics of OBGYNs and important items.

		Respondent’s sex (n = 88)		Years of experience as a OBGYN (n = 91)				Training on pregnant workers and the OProMa (n = 85)	
		Man (n = 31)	Woman (n = 57)	0–10 (n = 18)	11–20 (n = 31)	21–30 (n = 28)	31–45 (n = 14)	Yes (n = 43)	No (n = 42)
		% (n)	% (n)	% (n)	% (n)	% (n)	% (n)	% (n)	% (n)
Perceived knowledge about MPL	None at all	-	2 (1)	-	3 (1)	-	-	-	2 (1)
	Some	35 (11)	28 (16)	67 (12)	16 (5)	36 (10)	14 (2)	16 (7)	40 (17)
	Fairly good	52 (16)	61 (35)	28 (5)	74 (23)	50 (14)	64 (9)	65 (28)	52 (22)
	Very good	13 (4)	9 (5)	6 (1)	6 (2)	14 (4)	21 (3)	19 (8)	5 (2)
*p*-value		0.912		0.210				**0.003**	
Frequency at which OBGYNs asked for a risk analysis	Never/rarely	41 (12)	31 (16)	25 (4)	31 (9)	48 (13)	31 (4)	21 (9)	48 (20)
	Sometimes	31 (9)	38 (20)	31 (5)	38 (11)	33 (9)	59 (7)	44 (19)	31 (13)
	Often	10 (3)	15 (8)	25 (4)	14 (4)	4 (1)	8 (1)	14 (6)	12 (5)
	Nearly always/always	17 (5)	15 (8)	19 (3)	17 (5)	15 (4)	8 (1)	21 (9)	9 (4)
*p*-value		0.930		0.071				**0.022**	
Ask questions about satisfaction at work	Never/rarely	3 (1)	5 (3)	5 (1)	6 (2)	4 (1)	-	2 (1)	2 (1)
	Sometimes	34 (10)	25 (14)	39 (7)	32 (10)	22 (6)	15 (2)	21 (9)	33 (14)
	Often	34 (10)	18 (10)	28 (5)	32 (10)	22 (6)	15 (2)	32 (14)	21 (9)
	Nearly always/always	28 (8)	52 (29)	28 (5)	29 (9)	52 (14)	70 (9)	44 (19)	43 (18)
*p*-value		**0.038**		**0.005**				0.788	
Frequency of prescription of preventive leave during normal pregnancies	Never/rarely	42 (12)	35 (18)	38 (6)	34 (10)	48 (13)	15 (2)	33 (14)	41 (17)
	Sometimes	14 (4)	38 (20)	31 (5)	31 (9)	30 (8)	38 (59	30 (13)	36 (15)
	Often	34 (10)	13 (7)	12 (2)	24 (7)	15 (4)	31 (4)	16 (7)	21 (9)
	Nearly always/always	10 (3)	13 (7)	19 (3)	10 (3)	7 (2)	15 (2)	21 (9)	2 (1)
*p*-value		0.711		0.325				0.101	
Frequency of prescription of sick leave during normal pregnancies	Never/rarely	14 (4)	17 (9)	31 (5)	17 (5)	7 (2)	8 (1)	21 (9)	7 (3)
	Sometimes	27 (8)	27 (14)	13 (2)	28 (8)	33 (9)	30 (4)	37 (16)	19 (8)
	Often	45 (13)	35 (18)	56 (9)	34 (10)	30 (8)	54 (7)	28 (12)	52 (22)
	Nearly always/always	14 (4)	21 (11)	-	21 (6)	30 (8)	8 (1)	14 (6)	21 (9)
*p*-value		0.771		0.717				**0.004**	

The model simultaneously includes the sex of OBGYNs, their years of experience, and whether they participated in training on pregnant workers and the OProMa, adjusting for place of practice.

The frequency with which OBGYNs asked questions about their pregnant patient’s job satisfaction was associated with the respondent’s sex (women more likely than men, *p* = .038) and years of experience (more experienced were more likely, *p* = .005).

OBGYNs who had undergone training on pregnant workers and the OProMa believed that they had better knowledge of MPL (*p* = .003), were more likely to ask for an RA in cases of presumed occupational risks (*p* = .022), and were less likely to prescribe sick leave during non-pathological pregnancy when the patient face an occupational risk (*p* = .004).

Although the association did not reach the level of statistical significance, data showed that trained OBGYNs were more likely to prescribe preventive leave when non-pathological pregnancies faced a proven occupational risk. Indeed, 21% of OBGYNs who followed the course claimed that they “always” prescribed preventive leave, versus 2% of those who had not undergone the training.

### Hierarchical cluster analysis

#### Typology of practice

Our hierarchical cluster analysis identified four groups of OBGYNs having similar types of practices with regard to MPL. **Table 5** summarize the factors distinguishing these groups.

**Table 5:** Types of practice defined using hierarchical clusters analysis.

Variables used to cluster practices			Practices in line with legislation (n = 29)	Practices on a case-by-case basis (n = 39)	Limited practices in line with legislation (n = 12)	Limited and heterogeneous practices (n = 2)
			% (n)	% (n)	% (n)	% (n)
“Often” or “always” ask questions about:		Profession	100 (29)	100 (39)	100 (12)	50 (1)
		Occupational risks	90 (26)	90 (35)	83 (10)	-
		Workplace conditions	86 (25)	95 (37)	67 (8)	-
		Satisfaction at work	79 (23)	72 (28)	58 (7)	-
Frequency at which OBGYNs asked for an occupational risk analysis		Never/rarely	7 (2)	44 (17)	50 (6)	100 (2)
		Sometimes	41 (12)	38 (15)	33 (4)	-
		Often	17 (5)	10 (4)	17 (2)	-
		Nearly always/always	35 (10)	8 (3)	-	-
Contact with the employer of a patient whose work poses a risk to pregnancy			79 (23)	56 (22)	25 (3)	50 (1)
Reasons explaining no contact with employers in cases involving suspected occupational risk and the absence of a risk analysis		Patient refusal	59 (17)	51 (20)	8 (1)	50 (1)
		Time constraints	38 (11)	10 (4)	75 (9)	-
		Lack of experience or competencies	3 (1)	26 (10)	83 (10)	-
		Need for medical secrecy	7 (2)	21 (8)	-	50 (1)
		I did not think about it	10 (3)	15 (6)	25 (3)	-
		Occupational physician’s responsibility	3 (1)	23 (9)	25 (3)	50 (1)
Frequency of prescription of preventive leave during normal pregnancies		Never/rarely	10 (3)	44 (17)	59 (7)	100 (2)
		Sometimes	35 (10)	36 (14)	33 (4)	-
		Often	31 (9)	15 (6)	-	-
		Nearly always/always	24 (7)	5 (2)	8 (1)	-
Frequency of prescription of sick leave during normal pregnancies		Never/rarely	27 (8)	8 (3)	8 (1)	-
		Sometimes	48 (14)	20 (8)	17 (2)	-
		Often	17 (5)	52 (20)	42 (5)	100 (2)
		Nearly always/always	7 (2)	20 (8)	33 (4)	-
When OBGYNs prescribe sick leave instead of preventive leave it is “nearly always” or “always” because of:		A request by the patient	38 (11)	66 (26)	92 (11)	50 (1)
		A request by the employer	10 (3)	33 (13)	50 (6)	-
		Habit	3 (1)	39 (15)	84 (10)	50 (1)
		Time constraints	7 (2)	5 (2)	83 (10)	50 (1)
		A lack of competency	10 (3)	36 (14)	83 (10)	50 (1)
Frequency with which advice about MPL was given to patients		Never/rarely	10 (3)	10 (4)	17 (2)	-
		Sometimes	17 (5)	33 (13)	17 (2)	50 (1)
		Often	31 (9)	31 (12)	33 (4)	50 (1)
		Nearly always/always	42 (12)	26 (10)	33 (4)	-
Referred patients to occupational health physicians in cases involving suspected or proven occupational risks			83 (24)	67 (26)	-	50 (1)

*Practices in line with legislation*. This group of OBGYNs was identified based on following characteristics: they are more likely to ask for an RA (52%) and to contact the pregnant worker’s employer (79%) when they receive a patient whose job posed a risk to her pregnancy. In cases involving a non-pathological pregnancy and a proven occupational risk, more than half (55%) prescribe preventive leave in line with MPL. Finally, 83% of these OBGYNs state that they refer their patients to occupational physicians if there is a suspicion of an occupational risk.

*Practices on a case-by-case basis*. This group of OBGYNs was identified based on following characteristics: they ask for an RA less frequently (18%) and they contact employers less systematically (56%). In cases involving a non-pathological pregnancy and a proven risk, 71% “often/always” prescribe sick leave, versus 17% who “often/always” prescribe preventive leave. If there is a suspicion of an occupational risk, 67% refer their patients to an occupational physician. This group sometimes follow the MPL and sometimes did not.

*Limited practices in line with legislation*. This group of OBGYNs was identified based on following characteristics: the majority (83%) “rarely/never” ask for an RA, a minority (25%) state that they contact employers when there is a suspicion of an occupational risk. They evoke time constraints (75%) and a lack of competencies in the domain of occupational health (83%) for not contacting employers. In cases involving non-pathological pregnancies and a proven occupational risk, 75% “often/always” prescribe sick leave, versus 8% who “often/always” prescribe preventive leave. When this group prescribe sick leave rather than preventive leave, it was “often/always” at the patient’s request (92%), through habit (84%), because of time constraints (83%), and because of a lack of competencies in the occupational health area (83%). None of these OBGYNs refers their patients to an occupational physician when they suspect an occupational risk.

*Limited and heterogenous practices*. Hierarchical cluster analysis revealed two OBGYNs who responded differently from all the others. Their practice distanced itself from that advocated by the MPL. In cases involving non-pathological pregnancies yet proven occupational risks, they never ask for an RA or prescribe preventive leave. In such cases, they “often” prescribe sick leave. Because of the restricted number of OBGYNs in this cluster, a proper interpretation of their practice is difficult.

#### Associations with types of practices

**Table 6** shows the associations between types of practices, which were identified in preceding cluster analysis, and the variables which we considered to be “determinant” as well as the variables concerning OBGYNs attitudes vis-à-vis MPL and preventive leave. The different types of practice are associated neither with OBGYNs’ canton of practice nor with OBGYN’s place of practice (public vs private practice).

**Table 6:** Associations with types of practices.

			Practices in line with legislation (n = 29)	Practices on a case-by-case basis(n = 39)	Limited practices in line with legislation (n = 12)	Limited and heterogeneous practices (n = 2)
		*p*-value	% (n)	% (n)	% (n)	% (n)
Perceived knowledge about MPL	Not at all		-	3 (1)	-	-
	Some		10 (3)	33 (13)	42 (5)	100 (2)
	Fairly well		69 (20)	59 (23)	42 (5)	-
	Very good	**0.025**	21 (6)	5 (2)	16 (2)	-
OBGYNs who attended a training program on pregnant employees and the OProMa		0.089	69 (20)	41 (16)	42 (5)	50 (1)
MPL is an important means of protecting pregnant employees		0.171	100 (29)	100 (39)	92 (11)	100 (2)
MPL is too burdensome on employers		0.075	63 (19)	46 (18)	83 (10)	50 (1)
MPL is insufficient because it does not cover all female employees		0.519	90 (26)	85 (33)	100 (12)	100 (2)
MPL is insufficient because it does not cover all occupational risks		0.434	69 (20)	79 (31)	92 (11)	100 (2)
Prescribing preventive leave should be the responsibility of an occupational health physician		0.664	76 (22)	82 (32)	92 (11)	100 (2)
Prescribing preventive leave may adversely affect the patient, particularly on her return to work after maternity leave		0.428	86 (25)	79 (31)	92 (11)	50 (1)

There was a significant association between OBGYNs’ knowledge of MPL and their practice cluster. Of those OBGYNs whose practice was in line with legislation, 90% estimated that they knew the national MPL “well/very well" (*p* = .025). This percentage was weaker in the other groups, with 64% in the case-by-case group and 58% in the group whose practice was limited.

The 69% of OBGYNs who were applying MPL in line with the legislation had undergone training on the OProMa. Finally, 83% of the OBGYNs whose application of MPL in their practice was limited considered that these provisions are too burdensome on employers.

## Discussion

Several studies highlight negative effects of certain occupational exposures for the health of pregnant workers and for their future children. For example, two recent meta-analysis [10, 11], carried out on 80 studies (with a total of 853’149 women) show that physically demanding job expose pregnant workers to higher risks of adverse pregnancy outcomes, such as preterm delivery (<37 weeks’ of gestation), low-birth-weight (birth weight <2,500 grams), small-for-gestational-age (SGA, birth weight < 10th percentile for the gestational age), miscarriage (loss of the fetus prior to 20 weeks’ of gestation), gestational hypertension and pre-eclampsia.

Literature also shows the effectiveness of the right to benefit from MPL on preterm delivery and small-for-gestational-age children [8, 9]. In terms of human resources, in Norway, the study conducted by Kristensen, Nordhagen [41]indicated that job adjustment allowed a decreased of absenteeism by nearly 11% during pregnancy. Other studies pointed out that workplace adjustments and job reassignment may represent a step forward in order to reduce absenteeism among pregnant workers, notably through sick leaves [42]. In Spain, the study conducted by Villar, Serra [43]shows that “Pregnancy occupational risk” leave, allows pregnant workers to adequately benefit from a protection toward occupational risks, notably physical, safety, ergonomic and psychosocial risks. Moreover, in the study by Buzzanell and Liu [44], all of the employees who felt that they had been supported during their pregnancy returned to paid work with the same employers. On the contrary, half of the employees who did not feel supported by their organisation during their pregnancy left their place of work after their maternity leave.

Data showed that several aspects of OBGYNs’ implementation of MPL in their daily practice could be improved. Moreover, the legislation in itself deserves to be rethought.

1. The majority of OBGYNs thought they had good knowledge about MPL. They also claimed to be highly sensitive to issues surrounding their patients’ occupational activities during pregnancy consultations. Compared to previous studies in Switzerland, our findings show that OBGYNs’ understanding of MPL had grown [45]. Nevertheless, these results must be nuanced. Although the majority estimated that they knew the legislation well, only a minority applied it properly in their practice. Furthermore, numerous OBGYNs stated that they advised their patients about MPL, although only some of them estimated having a good knowledge of the legislation and applied it properly in practice. Their declarations suggest that they might overestimate their knowledge.
2. OBGYNs perceived the absence of RAs, and the fact that employers fail to provide them, to be one of the main reasons explaining the difficulties encountered in implementing the OProMa with employers. In Switzerland, an online questionnaire covering a stratified sample of employers showed similar deficiencies in RA: only 16% of companies interviewed stated that they had carried one out [28]. Yet the minority of OBGYNs stated that they asked for RAs when receiving patients whose jobs involved a risk to their pregnancy. Because of the significant lack of RAs in companies, a large number of pregnant employees continued to work in conditions which put their health and that of their unborn child at risk [46–48]. RAs represent an essential tool in the evaluation of the occupational risks faced by pregnant women and in the decision whether to prescribe preventive leave, thus the reasons why OBGYNs do not requests RA when convinced that their patients face an occupational risk merit further research, notably via qualitative interviews.
3. The majority of OBGYNs claimed to have encountered difficulties implementing the OProMa with employers. The principal reason for this was that employers asked for their employees to be prescribed sick leave, even when there was no medical diagnosis to justify this decision. We could interpret the high rate of sick leave among pregnant workers as a product of the Swiss social insurance system. With sick leave mostly financed by the employer’s loss-of-income insurance, employers may prefer pregnant women to take sick leave than spending money on adapting their workstations.
4. A large number of OBGYNs consider the current legislation as insufficient because of its failure to cover all workers and all occupational risks. Some working conditions that are perceived "at risk" by the OBGYNs (e.g. a detrimental psychological atmosphere or stressful job) do not fall into the predetermined categories stated in the OProMa. These situations legally justify neither a sick leave nor a preventive leave; OBGYNs may prescribe sick leave certificates as the only possible solution in order to protect their patients. These results suggest that the legislation in itself should be rethought and improved.
5. OBGYNs under-prescribed preventive leave and over-prescribed sick leave in cases of non-pathological pregnancies. The main reason mentioned which guides them in their decision was the patient’s request. These findings agree with those of certain Swedish studies [32, 49, 50] which found that sick leave was often requested by pregnant employees because of the perceived arduousness of their work. Given that the costs of sick leave are often covered by an employer’s loss-of-income insurance and that sick leave does not entail workplace’s adaptations, employees may well prefer this type of leave to remain on good terms with their employers, especially for when they return from maternity leave. Indeed, a recent study by Rudin, Stutz [28]showed that 10% of Swiss workers were threatened with being fired when they announced their pregnancy to their employer. Pregnancy itself can be a trigger for conflict with one’s employer.

It also seems that for over one third of OBGYNs, the decision to prescribe sick leave rather than preventive leave is merely a habit. The motivations underlying OBGYNs’ practices require further investigation, notably via qualitative interviews.

OBGYNs also evoked a perceived lack of competencies needed for them to prescribe preventive leave. Statements in the literature [31, 32] revealed that OBGYNs considered themselves to be *bad judges* when it came to evaluating the arduousness of their patients’ working conditions. Indeed, the majority of OBGYNs think that prescribing preventive leave should be the responsibility of occupational physicians. This raises the question whether it was appropriate for the Swiss legislation (OProMa) to confer the role of evaluating the need for preventive leave for pregnant employees on OBGYNs in the first place.

The format and content of current pregnancy consultations also deserve some considerations. These put a very strong focus on clinical and biomedical screening processes and are of limited duration (about 20–30 minutes). The lack of time to talk about or apply the practices recommended by Switzerland’s MPL was raised several times by OBGYNs.

### Strengths and limitations

So far, OBGYNs’ practices with regard to the implementation of MPL have been barely investigated. The relatively good response rate to the study questionnaire -nearly one third of those contacted answered it- demonstrated a significant interest in the theme from OBGYNs working in the French-speaking part of the country. The response rate obtained in our survey is in line with the average response rate generally obtained in online surveys of health professionals [51]. We can assume that our conclusions are valid throughout French-speaking Switzerland, since no association between the canton of practice and the outcomes was found.

Nevertheless, our findings do have certain limitations.

First, although the OProMa is a federal legislation, variations in local practice may exist. The extrapolation of our results to the rest of Switzerland (German-speaking side and Italian-speaking side) thus faces limitations. Therefore, studies that fully cover the Swiss context should be carried out. However, the deficiencies revealed by the present study and the perspectives presented may provide practical suggestions as well as some interesting thoughts in contexts where OBGYNs are directly involved in MPL implementation. Second, questionnaire research is very dependent on the accuracy of answers provided by the participants [52]. Given that study participation was voluntary, we cannot exclude a positive selection bias in our sample. Moreover, by assuming that the OBGYNs who responded to the survey are those most interested and more sensitive to the relation between pregnancy issues and working conditions, our results may present an over-favourable view of the reality. In addition, the self-reported format of the questionnaire could introduce a social desirability bias.

Our results help to identify OBGYNs' actual practices regarding the application of MPL and show some indirect indicators of how legislation is implemented in the companies. Hierarchical cluster analysis allows to observe the diversity of OBGYN practices. Considering that only a minority of OBGYNs act in accordance with the legislation (Group 1: Practices in line with legislation, see **Table 5**), it may be concluded that some pregnant workers cannot benefit from protective measures as recommended by the legislation.

## Conclusion and perspectives

These results demonstrate the need to improve the implementation of maternity protection legislation (MPL) among companies in Switzerland. Results also demonstrate that legislation itself should be improved: it does not cover all the risks perceived by the OBGYNs (e.g. the detrimental psychological atmosphere in the workplace or stressful job). Moreover, gynecologist-obstetricians (OBGYNs) seem to have difficulty taking up the essential role which Swiss legislation has conferred upon them, namely the prescription of preventive leave to pregnant employees facing an occupational risk. One solution to help OBGYNs be clear about the action they should take with regard to pregnant workers might be a redefinition of the roles set out in Switzerland’s Ordonnance on Maternity Protection (OProMa). Associating occupational health physicians more closely with decisions about preventive leave has been suggested. However, this should be done by ensuring that both OBGYNs and pregnant workers have adequate resources (economic, time) to effectively benefit from consultation with the occupational physician. Furthermore, Switzerland is characterized by a great shortage of occupational physicians, which makes it difficult to exploit this resource. Since 2015, the Occupational Health and Environment Department of Center for Primary Care and Public Health (Unisanté) has set up a specialized consultation for pregnant workers, referred by their OBGYN. This consultation, carried out by occupational health physicians, was developed to evaluate the suitability of the workplace for pregnant workers, to inform the workers and their employers of their rights and duties and to help the company in the process of risk analysis and adjustment of workstations. This consultation is the only one in Switzerland. The wide use of this consultation by the OBGYNs informs about the need to be supported and advised by a specialist.

The data also showed positive effects of training about the OProMa on OBGYNs knowledge and the application of MPL. Thus, encouraging awareness of and systematic participation in training courses about pregnant employees and the OProMa could be a positive way forward. Furthermore, interdisciplinary research combining quantitative and qualitative data would be required. Moreover, because pregnancy at work involves complex issues the points of view of the various stakeholders should be sought and compared.

Finally, measures should be taken to avoid pregnancy-based discrimination or conflicts in the workplace, so that workers do not fear to exert their rights to job accommodations or preventive leave. Acting on OBGYNs’ practices by aligning them with the directives expressed in the legislation is important. However, it must be recognized that their practices reflect dilemmas and contradictions intrinsically linked to the current protection policies. Legal protection against dismissal and discrimination could be strengthened. The context and the social insurance system of maternity protection at work should also evolve, for example, by mutualising costs for companies, e.g. through the establishment of a legal prenatal leave or a fund for pooling the costs linked to workplace adjustments and, if necessary, preventive leave.

## Supporting information

S1 Dataset This is the raw dataset (French). (XLSX)

S2 Dataset This is the translated dataset (English). Canton of practice and age have been removed in order to preserve OBGYNs data confidentiality.
(PDF)

S1 Data Questionnaire provided to OBGYNs (French). (PDF)

S2 Data Translated version to the questionnaire provided to OBGYNs (English). (PDF)
