# Supplementary material for: Implementation of maternity protection legislation: Gynecologists’ perceptions and practices in French-speaking Switzerland
Source: PLoS One. 2020 Apr 30;15(4):e0231858. doi: 10.1371/journal.pone.0231858 (PMC7192633; doi:10.1371/journal.pone.0231858)
Supplement: S2 Dataset — Canton of practice and age have been removed in order to preserve OBGYNs data confidentiality. (PDF) [file pone.0231858.s002.pdf]

Information about yourself

I. In which canton do you mainly work? (one reply only, please)

- ☐ Fribourg, FR
- ☐ Geneva, GE
- ☐ Jura, JU
- ☐ Neuchâtel, NE
- ☐ Vaud, VD
- ☐ Valais, VS

II. How many years in total have you been practising as a gynaecologist–obstetrician?

_____ years

III. Sex

- ☐ Male
- ☐ Female

IV. In what year were you born? _____

Introduction

1. Are pregnancy consultations a part of your professional activity?

- ☐ No *(If you do not carry out pregnancy consultations, please do not fill out this questionnaire. Because this research project aims to understand gynaecologists' practices with regard to caring for pregnant employees in French-speaking Switzerland, pregnancy consultations must be a part of participants' professional activities.)*
- ☐ Yes

Work and pregnancy

2. Do you know the legal provisions for maternity protection, such as Switzerland's Ordinance on Maternity Protection at Work (OProMa)?

- ☐ Not at all
- ☐ Somewhat
- ☐ Fairly well
- ☐ Very well

3. When monitoring your pregnant patients, do you ask them questions about:

	Never or rarely	Sometimes	Often	Nearly always or always
Their profession.	☐	☐	☐	☐
The existence of any risks to their pregnancy in their workplace (load lifting, vibrations, exposure to chemical products, ionising radiation, noise, stress, etc.).	☐	☐	☐	☐
Their working conditions (e.g. hours, premises, interpersonal relationships, etc.).	☐	☐	☐	☐
Their job satisfaction.	☐	☐	☐	☐

4. What percentage of the pregnant patients whom you have consulted presented with an occupational risk to them or their unborn child?
___/100

5. What percentage of the pregnant patients whom you have consulted, whose job involved risks according to the OProMa, provided you with a risk analysis?
___/100

6. **In the list below, which 5 of these tasks which pose a risk to pregnant employees do you encounter most frequently among your patients?** Please tick the five most frequently encountered.

I never ask the question, so I cannot reply	☐
Moving heavy loads	☐
Awkward movements and postures	☐
Prolonged standing (e.g. a crouched position or one with frequent or prolonged bending forward).	☐
Restrictive work schedules (too long (> 9 h per day), shift work, night work, etc.).	☐
Harmful psychological climate (e.g. poor relationship with a superior, patient is a victim of bullying, etc.)	☐
Stressful work	☐
Exposure (potential) to micro-organisms	☐
Exposure (potential) to chemical products which are a danger to the foetus	☐
Exposure to noise	☐
Exposure to vibrations (e.g. using tools or operating machinery)	☐
Exposure to cold or heat	☐
Exposure (potential) to ionising radiation	☐
Exposure (potential) to non-ionising radiation	☐
Timed work or assembly line work	☐
Work in a pressurised environment and/or O_2 depleted rooms	☐

Caring for working pregnant patients

7. When you consult a pregnant patient whose job poses a risk to her pregnancy, do you ask for a risk analysis (as recommended by OProMa)?

- ☐ Never/rarely
- ☐ Sometimes
- ☐ Often
- ☐ Nearly always/always

8. Have you ever contacted the employer (or line manager) of a pregnant patient whose work poses a risk to her pregnancy?

- ☐ No (go straight to question 12)
- ☐ Yes

9. Have you ever contacted an employer …

	Never or rarely	Sometimes	Often	Nearly always or always
To inform them of their responsibilities in accordance with OProMa?	☐	☐	☐	☐
To ask questions about the nature of the patient's work and any occupational risks?	☐	☐	☐	☐
To ask them whether they have had a risk analysis carried out by an occupational health physician or an occupational health and safety specialist?	☐	☐	☐	☐
To find a solution enabling changes to be made to the patient's workstation or working hours, etc.?	☐	☐	☐	☐

10. Have you ever had any difficulties communicating with employers? (more than one answer is possible)

- ☐ No, not especially
- ☐ Yes, because I did not have the time
- ☐ Yes, because the employer was unavailable
- ☐ Yes, because of medical secrecy issues

11. Have you ever had any difficulties with employers regarding the implementation of OProMa?

- ☐ No (go straight to question 12)
- ☐ Yes

11.1. If yes, why? (more than one answer is possible)

- ☐ A lack of cooperation on behalf of the employer
- ☐ The employer under-estimated the occupational risks involved
- ☐ The employer was unaware of their obligation to pay 80% of the pregnant employee's salary in case of a preventive leave
- ☐ The absence of a risk analysis carried out by an occupational health physician or an occupational health and safety specialist
- ☐ The employer asked me to put the pregnant worker on sick leave rather than write a certificate for preventive leave
- ☐ The employer claimed that they had financial difficulties

12. If you have identified a dangerous or arduous activity according to the provisions of OProMa, but you have not received a risk analysis, under what circumstances would you not contact the employer? (more than one answer is possible)

- ☐ If the patient did not want me to
- ☐ If I did not have the time
- ☐ If I did not have the experience or skills
- ☐ Out of a duty to medical secrecy
- ☐ I have never thought about it
- ☐ It is the occupational health physician's role rather than mine to look after my patients' occupational problems

13. In cases involving a <u>normal pregnancy</u> but <u>arduous professional and/or dangerous activities</u> according to OProMa, how often do you write out a <u>certificate for preventive leave</u> from the workstation?

Note that this is not sick leave but a disparity between the workstation and protection of the worker's health.

- ☐ Never/rarely
- ☐ Sometimes
- ☐ Often
- ☐ Nearly always/always

14. In cases involving a <u>normal pregnancy</u> but <u>arduous professional and/or dangerous activities</u> according to OProMa, how often do you <u>prescribe sick leave</u>?

- ☐ Never/rarely
- ☐ Sometimes
- ☐ Often
- ☐ Nearly always/always

15. What would make you prescribe sick leave rather than write out a certificate for preventive leave?

	Never or rarely	Sometimes	Often	Very often
A request by the patient	☐	☐	☐	☐
A request by the employer	☐	☐	☐	☐
That is how I normally proceed	☐	☐	☐	☐
I do not have time to write out certificates for preventive leave	☐	☐	☐	☐
I do not feel that I have the necessary skills to write out certificates for preventive leave	☐	☐	☐	☐

16. Do you give your patient's advice on the legal provisions surrounding maternity protection?

- ☐ Never/rarely
- ☐ Sometimes
- ☐ Often
- ☐ Nearly always/always

Difficulties encountered and resources at your disposal

17. How far would you agree with statements in the following list?

	Strongly disagree	Disagree	Agree	Strongly agree
The legal provisions for maternity protection are important instruments for the protection of pregnant workers.	☐	☐	☐	☐
The legal provisions for maternity protection are too onerous on employers (e.g. risk analyses, payment of 80% of salary during preventive leave and for a medical consultation on workstation suitability, workstation adaptations, reassignment, etc.).	☐	☐	☐	☐
The legal provisions are insufficient because they do not cover all workers (e.g. domestic workers, the self-employed).	☐	☐	☐	☐
The legal provisions are insufficient because they do not cover all types of occupational risk (e.g. psychosocial risks).	☐	☐	☐	☐
Writing a certificate for preventive leave should be a job for the occupational health physician.	☐	☐	☐	☐
A certificate for preventive leave may risk harming the patient, particularly on her return from maternity leave (e.g. risk of being laid off).	☐	☐	☐	☐
Following the announcement of their pregnancy, some patients report tensions with their employer.	☐	☐	☐	☐
Some patients under-estimate the risks linked to their work.	☐	☐	☐	☐
Some patients exaggerate the risks linked to their work.	☐	☐	☐	☐

18. When you suspect or discover an occupational risk, do you ever direct your pregnant employee patient to an occupational health physician?

☐ No (go straight to question 20)

☐ Yes (go to question 21 after having answered question 19)

19. If yes, why do you refer the patient to an occupational health physician when you suspect or discover occupational risks? (more than one answer is possible)

- ☐ For them to manage the situation because I do not have the time
- ☐ For them to manage the situation because I do not have the skills
- ☐ To get recommendations on the patient's aptitude to work at her workstation
- ☐ To carry out a risk analysis of the pregnant worker's workstation
- ☐ To protect myself legally in case of any dispute

20. If not, why don't you orient the pregnant employee towards an occupational physician if you suspect or discover occupational risks? (more than one answer is possible)

- ☐ Because I do not have the time to orient them
- ☐ Because issues regarding pregnant women's professional lives are not a priority in my practice
- ☐ Because I could not find an occupational physician available
- ☐ Because I do not know any occupational physicians
- ☐ Because I can manage the situation myself
- ☐ Because I have never thought about it

Conclusion

21. Have you ever:

21.1. Had any <u>training</u> on pregnant employees and OProMa?

- ☐ Yes
- ☐ No

21.2. Were these <u>training courses or sessions</u> useful in your practice?

- ☐ Yes
- ☐ No

22. Please write down any comments, suggestions, thoughts, etc. that you would like to inform us about?

____
____
____
____
____
____

Thank you for participating in this survey!

If you wish to be informed about the results of this research project, please send an email to secretariat.medecine@i-s-t.ch and simply write "I wish to be informed about the results of the OProMa FNS project" in the subject line (your email requesting information will be treated separately from the data collection and analyses parts of our project).

We sincerely thank you for having spent the time to contribute to this research project aimed at improving the management and care of pregnant employees and their unborn children.
