# Supplementary material for: Implementation of maternity protection legislation: Gynecologists’ perceptions and practices in French-speaking Switzerland
Source: PLoS One. 2020 Apr 30;15(4):e0231858. doi: 10.1371/journal.pone.0231858 (PMC7192633; doi:10.1371/journal.pone.0231858)
Supplement: S1 Data — (PDF) [file pone.0231858.s003.pdf]

Informations vous concernant

I. Dans quel canton exercez-vous principalement? (une seule réponse svp)

- ☐ FR
- ☐ GE
- ☐ JU
- ☐ NE
- ☐ VD
- ☐ VS

II. Depuis combien d'années cumulées exercez-vous la gynécologie-obstétrique?

……. ans

III. Êtes-vous…

- ☐ un homme
- ☐ une femme

IV. Quelle est votre année de naissance? ____

Introduction

1. Est-ce que les consultations de grossesse font partie de votre activité?

☐ Non *(Si vous ne faites pas de consultations de grossesse merci de ne pas remplir ce questionnaire. En effet, comme notre recherche vise à comprendre les pratiques des gynécologues concernant la prise en charge des travailleuses enceintes en Suisse romande, il est nécessaire que les consultations de grossesse fassent partie de l'activité professionnelle des participants).*

☐ Oui

Travail et grossesse

2. Connaissez-vous les dispositifs juridiques de la protection de la maternité (comme la Loi sur le Travail et l'Ordonnance sur la Protection de la Maternité (OProMa))?

☐ Pas du tout

☐ Un peu

☐ Assez bien

☐ Très bien

3. Pour le suivi de vos patientes enceintes, posez-vous des questions sur :

	Jamais ou rarement	Parfois	Souvent	Presque toujours ou toujours
Leur profession.	☐	☐	☐	☐
L'existence à la place de travail, de risques pour la grossesse (tels que manutention, vibrations, exposition à des produits chimiques, à des radiations ionisantes, au bruit, au stress, etc.).	☐	☐	☐	☐
Les conditions de travail (p. ex. horaires, locaux, climat relationnel, etc.).	☐	☐	☐	☐
Leur satisfaction au travail.	☐	☐	☐	☐

4. Sur 100 patientes enceintes vues en consultation, combien, selon vous, ont un travail à risque pour elles-mêmes et/ou leur enfant à naître?

___/100

5. Sur 100 patientes enceintes vues en consultation ayant un travail à risque selon l'OProMa, combien ont eu une analyse de risques qui vous a été fournie?

___/100

6. **Dans la liste ci-dessous, quelles sont les 5 tâches à risque pour la grossesse que vous rencontrez le plus fréquemment chez vos patientes?** Cochez les 5 plus fréquentes svp.

Je ne pose jamais la question donc je ne peux pas répondre.	☐
Déplacement de charges lourdes.	☐
Mouvements et postures contraignants.	☐
Station debout prolongée (p. ex. position accroupie ou penchée en avant fréquente et prolongée).	☐
Horaire contraignant (trop long (> 9h/j), irrégulier, travail de nuit, etc.).	☐
Climat psychologique délétère (p. ex. mauvaise entente avec un supérieur, patiente victime de ”mobbing”, etc.).	☐
Travail stressant.	☐
Exposition (potentielle) à des microorganismes.	☐
Exposition (potentielle) à des produits chimiques dangereux pour le fœtus.	☐
Exposition au bruit.	☐
Exposition aux vibrations (p. ex. usage d’outils ou conduite d’engins).	☐
Exposition au froid ou au chaud.	☐
Exposition (potentielle) aux radiations ionisantes.	☐
Exposition (potentielle) aux radiations non ionisantes.	☐
Travail cadencé à la chaîne.	☐
Travail en surpression et/ou locaux appauvris en O_2.	☐

Prise en charge des patientes travailleuses enceintes

7. **Lorsque vous recevez une patiente enceinte dont le travail est à risque pour sa grossesse, demandez-vous l'analyse de risques (telle que préconisée dans l'OProMa) ?**

- ☐ Jamais ou rarement
- ☐ Parfois
- ☐ Souvent
- ☐ Presque toujours ou toujours

8. **Vous est-il déjà arrivé de prendre contact avec l'employeur (ou le responsable hiérarchique) d'une patiente enceinte dont le travail était à risque pour sa grossesse?**

- ☐ Non (passer à la question 12)
- ☐ Oui

9. **Avez-vous déjà pris contact avec un employeur…**

	Jamais ou rarement	Parfois	Souvent	Presque toujours ou toujours
Pour l'informer de sa responsabilité découlant de l'OProMa?	☐	☐	☐	☐
Pour l'interroger sur la nature du travail et de certains risques professionnels?	☐	☐	☐	☐
Pour lui demander s'il a fait effectuer une analyse de risques par un médecin du travail ou un spécialiste de la santé sécurité au travail?	☐	☐	☐	☐
Pour trouver une solution permettant d'adapter le poste de travail, aménager des horaires, etc.?	☐	☐	☐	☐

10. **Avez-vous rencontré des difficultés à contacter les employeurs?** (plusieurs réponses possibles)

- ☐ Non, pas spécialement.
- ☐ Oui, par manque de temps de ma part.
- ☐ Oui, par indisponibilité de la part de l'employeur.
- ☐ Oui, en raison du secret médical.

11. Avez-vous rencontré des difficultés avec les employeurs dans la mise en œuvre de l'OProMa?

- ☐ Non (passer à la question 12)
- ☐ Oui

11.1 Si oui, pour quelles raisons? (plusieurs réponses possibles)

- ☐ Manque de collaboration de l'employeur.
- ☐ L'employeur sous-estime les risques professionnels.
- ☐ Méconnaissance de l'employeur de son obligation de payer 80% du salaire en cas d'inaptitude de la travailleuse enceinte.
- ☐ Absence d'une analyse de risques effectuée par un médecin du travail ou un spécialiste de la santé sécurité au travail (MSST).
- ☐ L'employeur me demande un arrêt de travail plutôt qu'un avis d'inaptitude.
- ☐ L'employeur invoque des difficultés économiques.

12. Si vous avez identifié une activité dangereuse ou pénible selon l'OProMa et que vous n'avez pas l'analyse de risques, pourquoi renonceriez-vous à contacter l'employeur? (plusieurs réponses possibles)

- ☐ Par refus de la patiente.
- ☐ Par manque de temps.
- ☐ Par manque d'expérience et de compétence.
- ☐ Par respect du secret médical.
- ☐ Je n'y ai pas pensé.
- ☐ Ce n'est pas à moi de m'occuper des problématiques de travail de mes patientes mais plutôt au médecin du travail.

13. En cas de <u>grossesse physiologique</u> et d'activités <u>professionnelles pénibles et/ou dangereuses</u> selon l'OProMa, à quelle fréquence réalisez-vous un <u>certificat d'avis d'inaptitude</u> au poste de travail?

Attention, il ne s'agit pas d'un arrêt de travail mais d'une inadéquation entre le poste du travail et la protection de la santé.

- ☐ Jamais ou rarement
- ☐ Parfois
- ☐ Souvent
- ☐ Presque toujours ou toujours

14. En cas de grossesse physiologique et d'activités professionnelles pénibles et/ou dangereuses selon l'OProMa, à quelle fréquence réalisez-vous un certificat d'incapacité de travail (= arrêt de travail)?

- ☐ Jamais ou rarement
- ☐ Parfois
- ☐ Souvent
- ☐ Presque toujours ou toujours

15. Qu'est-ce qui vous amène à rédiger un certificat d'incapacité de travail (= arrêt de travail) plutôt qu'un certificat d'avis d'(in)aptitude?

	Jamais ou rarement	Parfois	Souvent	Très souvent
La demande de la patiente.	☐	☐	☐	☐
La demande de l'employeur.	☐	☐	☐	☐
J'ai l'habitude de procéder ainsi.	☐	☐	☐	☐
Je n'ai pas le temps de faire l'avis d'(in)aptitude.	☐	☐	☐	☐
Je ne me sens pas la compétence nécessaire à rédiger l'avis d'inaptitude.	☐	☐	☐	☐

16. Donnez-vous des conseils sur les dispositifs juridiques de la protection de la maternité à vos patientes ?

- ☐ Jamais ou rarement
- ☐ Parfois
- ☐ Souvent
- ☐ Presque toujours ou toujours

Difficultés rencontrées et ressources

17. Dans quelle mesure êtes-vous d'accord avec les affirmations suivantes?

	Pas du tout d'accord	Pas d'accord	D'accord	Tout à fait d'accord
Les dispositifs juridiques de protection de la maternité sont des outils importants pour la protection de la travailleuse enceinte.	☐	☐	☐	☐
Les dispositifs de protection de la maternité sont trop lourds pour les employeurs (p. ex. analyse de risques, paiement du salaire (80%) en cas d'avis d'inaptitude et de la consultation médicale d'avis au poste de travail, adaptation du poste, reclassement, etc.).	☐	☐	☐	☐
La réglementation est insuffisante car elle ne couvre pas toutes les travailleuses (p. ex. les employées domestiques, les indépendantes).	☐	☐	☐	☐
La réglementation est insuffisante car elle ne couvre pas tous les risques professionnels (p. ex. les risques psycho-sociaux).	☐	☐	☐	☐
L'avis d'aptitude devrait relever du médecin du travail.	☐	☐	☐	☐
L'avis d'inaptitude risque de nuire à la patiente, en particulier au retour du congé maternité (p. ex. risque de licenciement).	☐	☐	☐	☐
Depuis l'annonce de leur grossesse, certaines patientes rapportent des tensions avec leur employeur.	☐	☐	☐	☐
Certaines patientes sous-estiment les risques liés à leur travail.	☐	☐	☐	☐
Certaines patientes exagèrent les risques liés à leur travail.	☐	☐	☐	☐

18. Lorsque vous soupçonnez ou constatez des risques professionnels, vous arrive-t-il d'adresser votre patiente travailleuse enceinte à un médecin du travail?

☐ Non (passer à la question 20)

☐ Oui (passer à la question 21, après avoir répondu à la question 19)

19. Si oui, pourquoi adressez-vous la patiente au médecin du travail lorsque vous soupçonnez ou constatez des risques professionnels? (plusieurs réponses possibles)

- ☐ Pour gérer la situation car je n'ai pas le temps.
- ☐ Pour gérer la situation car je ne suis pas compétent.
- ☐ Pour obtenir des recommandations sur l'avis d'aptitude au poste de travail.
- ☐ Pour effectuer l'analyse de risques du poste de travail de la travailleuse enceinte.
- ☐ Pour me couvrir légalement en cas de contestation.

20. Si non, pourquoi n'orientez-vous pas la travailleuse enceinte à un médecin du travail alors que vous soupçonnez ou constatez des risques professionnels? (plusieurs réponses possibles)

- ☐ Parce que je n'ai pas le temps de l'orienter.
- ☐ Parce que la question professionnelle des femmes enceintes n'est pas prioritaire dans ma pratique.
- ☐ Parce que je n'ai pas trouvé de médecin du travail disponible.
- ☐ Parce que je ne connais pas de médecin du travail.
- ☐ Parce que je peux gérer la situation de façon autonome.
- ☐ Parce que je n'y ai jamais pensé.

Conclusion

21. Avez-vous bénéficié :

21.1 d'une <u>formation</u> sur la travailleuse enceinte et l'OProMa?

- ☐ Oui
- ☐ Non

21.2 Cette/ces <u>formation(s)</u> était-elle/étaient-elles utile(s) pour votre pratique?

- ☐ Oui
- ☐ Non

22. Avez-vous des commentaires, suggestions, réflexions, etc. à nous communiquer?

____
____
____
____
____
____

Merci pour votre participation à cette enquête !

Si vous souhaitez être informée des résultats de cette recherche, nous vous remercions de nous envoyer un mail au secretariat.medecine@i-s-t.ch en indiquant simplement en objet « Souhaite être informé des résultats OProMa FNS » (cet e-mail sera traité séparément de l’analyse des résultats).

Nous vous remercions vivement de nous avoir consacré du temps et de contribuer à cette recherche afin d’améliorer la prise en charge des travailleuses enceintes et de leurs enfants à naître.
